# Association between serum neuron-specific enolase, age, overweight, and structural MRI patterns in 901 subjects

**DOI:** 10.1038/s41398-017-0035-0

**Published:** 2017-12-08

**Authors:** Johanna Hoffmann, Deborah Janowitz, Sandra Van der Auwera, Katharina Wittfeld, Matthias Nauck, Nele Friedrich, Mohamad Habes, Christos Davatzikos, Jan Terock, Martin Bahls, Annemarie Goltz, Angela Kuhla, Henry Völzke, Hans Jörgen Grabe

**Affiliations:** 1 Department of Psychiatry and Psychotherapy, University Medicine Greifswald, Greifswald, Germany; 2 German Center for Neurodegenerative Diseases (DZNE), Site Rostock, Greifswald, Germany; 3 Institute of Clinical Chemistry and Laboratory Medicine, University Medicine Greifswald, Greifswald, Germany; 4 DZHK (German Centre for Cardiovascular Research), Partner site Greifswald, Greifswald, Germany; 5 Institute for Community Medicine, University Medicine Greifswald, Greifswald, Germany; 6 Department of Radiology, Section of Biomedical Image Analysis, University of Pennsylvania, Philadelphia, PA USA; 7 Department of Psychiatry and Psychotherapy, University Medicine Greifswald, HELIOS Hospital Stralsund, Stralsund, Germany; 8 Department of Internal Medicine B, University Medicine Greifswald, Greifswald, Germany; 9 Institute for Experimental Surgery, Rostock University Medical Center, Rostock, Germany; 10 DZD (German Centre for Diabetes Research), Site Greifswald, Greifswald, Germany

## Abstract

Serum neuron-specific enolase (sNSE) is considered a marker for neuronal damage, related to gray matter structures. Previous studies indicated its potential as marker for structural and functional damage in conditions with adverse effects to the brain like obesity and dementia. In the present study, we investigated the putative association between sNSE levels, body mass index (BMI), total gray matter volume (GMV), and magnetic resonance imaging-based indices of aging as well as Alzheimer’s disease (AD)-like patterns. Subjects/Methods: sNSE was determined in 901 subjects (499 women, 22–81 years, BMI 18–48 kg/m^2^), participating in a population-based study (SHIP-TREND). We report age-specific patterns of sNSE levels between males and females. Females showed augmenting, males decreasing sNSE levels associated with age (males: *p* = 0.1052, females: *p* = 0.0363). sNSE levels and BMI were non-linearly associated, showing a parabolic association and decreasing sNSE levels at BMI values >25 (*p* = 0.0056). In contrast to our hypotheses, sNSE levels were not associated with total GMV, aging, or AD-like patterns. Pathomechanisms discussed are: sex-specific hormonal differences, neuronal damage/differentiation, or impaired cerebral glucose metabolism. We assume a sex-dependence of age-related effects to the brain. Further, we propose in accordance to previous studies an actual neuronal damage in the early stages of obesity. However, with progression of overweight, we assume more profound effects of excess body fat to the brain.

## Introduction

The global prevalence of obesity has more than doubled since 1980. In 2014, >1.9 billion adults were overweight (body mass index (BMI) ≥25 kg/m²), from which over 600 million were obese (BMI ≥30 kg/m²)^1^. The global world population is aging and gaining weight; therefore, we will face the problem of many overweight elderly people in the future. There is growing evidence that the consequences of excess body weight extend to the brain. Specifically, human^2^ and animal studies^3^ reported effects of obesity on neuronal structure and function.

For example, obesity has been associated with increased gray matter (GM) damage^4–6^. However, the mechanism of how obesity, GM reductions, and cognitive alterations are related remains subject of ongoing discussion. Many studies reported midlife obesity to increase the risk for cognitive impairment^2^, and neurodegenerative diseases like Alzheimer’s disease (AD)^7^. Obesity accelerates neurodegenerative processes known in the aging brain^8^, and initiates structural cerebral alterations that result in impairments of memory performance in aging^5^.

Recent studies established indices that acquire cerebral atrophy patterns. They allow to distinguish between age-dependent atrophy (spatial pattern of atrophy for recognition of brain aging, SPARE-BA), and atrophy specifically found in clinically diagnosed AD cases (spatial patterns of abnormality for recognition of early AD, SPARE-AD)^9^ from magnetic resonance (MR) images. The SPARE-AD index is predictive for transition from normal cognition to mild cognitive impairment^10^, and further on to AD^11^. Habes et al.^9^ demonstrated that waist circumference (WC) was associated with more advanced brain aging patterns in a male sub-cohort in our study population.

In order to give evidence for the presence and extent of cerebral damage and alterations, we need brain-specific markers. In this context, there is growing evidence, that the neuron-specific enolase (NSE) could be a good candidate. NSE is an enzyme of the glycolytic pathway and is closely related to the differentiated state of mature nerve cells^12^. In neurons, NSE is primarily localized in the cytoplasm. Since NSE cannot be secreted by cells, an increase of NSE in cerebrospinal fluid (CSF) or serum is a marker for neuronal damage^13^. Using the Allen Brain Atlas, it was shown that NSE gene expression is increased in frontal and parietal lobes, claustrum, and cerebellum, with claustrum and cerebellum showing the highest gene expression^14^.

Previous studies demonstrated that different regions are affected by GM reduction in normal aging or AD. Cerebral alterations in aging or AD can be distinguished by SPARE-BA or SPARE-AD^9^, but no study investigated the association between aging patterns and serum neuron-specific enolase (sNSE) levels so far.

In addition of the effects of aging on GM, an increased body weight influences gray matter volume (GMV)^4^. First evidence of a putative link between obesity-associated alterations in GMV and sNSE levels was established by the study of Mueller et al.^14^. The authors described an inverse correlation between sNSE and GM density in hippocampal and cerebellar regions in overweight young adults. Therefore, we sought to investigate if sNSE levels would be indicative of these alterations in GMV associated with obesity in our population-based sample.

Additionally, medication may interfere with measurements of cerebral volume and function. Therefore, we controlled calculations also for antihypertensive and lipid-lowering drugs.

Based on findings in literature, we hypothesized, that

1. sNSE levels are positively associated with age,
2. sNSE levels are positively associated with increasing BMI or WC,
3. sNSE levels are negatively associated with GMV and structural aging patterns (SPARE-BA and SPARE-AD).

## Materials and methods

### Participants

We collected data in course of the population-based Study of Health in Pomerania (SHIP)^15–17^. The study comprises adult residents living in three cities and 29 communities, with a total population of 212,157. SHIP-TREND contains a stratified random sample of 8,016 adult Caucasians aged 20–79 years (baseline). Effectively, a total number of 4,420 subjects participated in the study. Baseline information was collected between 2008 and 2011. The only exclusion criterion for SHIP-TREND was participation in the parallel running study SHIP-0. The data of sNSE were collected within a subsample of SHIP-TREND, consisting of 1,000 participants without clinical diabetes mellitus.

The study was approved by the ethics committee of the University of Greifswald adhering to the Declaration of Helsinki. All subjects provided written informed consent.

### Clinical examination and medication

Clinical examination procedures have been described previously^4^.

Smoking was defined as current smoking (occasional; 1–14 cigarette(s) per day; ≥15 cigarettes per day), former smoking (occasional; 1–14 cigarette(s) per day; ≥15 cigarettes per day), and never smoking.

Current medication was recorded using Anatomical Therapeutic Chemical classification codes^18^. For our analysis, we used information on antihypertensive medication (C02*, C03*, C07*, C08*, and C09*) and lipid-lowering drugs (C10*).

### Laboratory analyses

Fasting blood samples (fasting ≥8 h) were drawn between before noon from the cubital vein of subjects in the supine position and analyzed immediately or stored at −80 °C. Serum concentrations of sNSE were determined using an immunoassay (cobas e 411 analyzer, Roche Diagnostics GmbH, Mannheim, Germany) with a functional sensitivity of 0.05 µg/L. The interassay variation was 4.4%.

Photometry was used to quantify high-density lipoprotein cholesterol (HDL-C) concentrations (Hitachi 704, Roche Diagnostics). Comparability in the longitudinal HDL-C analyses was ensured by using baseline HDL-C concentrations as the reference, and calculating corrected follow-up HDL-C concentrations based on a previous published conversion formula (HDL_fu_corr = −80 + (1.158 × HDL_fu))^19^. We quantified serum low-density lipoprotein (LDL-C) by applying a precipitation procedure using dextran sulphate (Immuno, Heidelberg, Germany) on an Epos 5060 (Eppendorf, Hamburg, Germany). LDL-C, HDL-C, and total cholesterol were measured in mmol/L as dimensional scores.

The laboratories analysing the samples of SHIP, participate in the official German external quality proficiency testing programmes. As often as available, all assays were calibrated against the international reference preparations.

### Magnetic resonance imaging

We asked participants to undergo whole-body magnetic resonance imaging (MRI). The image acquisition parameters of the whole-body MRI scans in SHIP have been described previously^20^. The images were acquired with 1.5 Tesla scanner (Magnetom Avanto: Siemens Medical Solutions, Erlangen, Germany). MRI images were available for all 901 subjects with information on sNSE. Subjects who fulfilled exclusion criteria against MRI (e.g., pregnancy, cardiac pacemaker), who refused participation and subjects with a stroke, Parkinson’s disease, epilepsy, hydrocephalus, enlarged ventricles, pathological lesions, history of cerebral tumor, and multiple sclerosis were excluded from the present analyses. Moreover, we excluded images with severe inhomogeneities of the magnetic field, strong movement artefacts, and images, who failed for quality control. Within the voxel based morphometry (VBM), 8 toolbox homogeneity check was conducted. After exclusion, 832 subjects remained in the sample (Supplementary Fig. 4). For detailed information for exclusion criteria of MRI of the brain in SHIP-TREND, see Supplementary Fig. 5.

For structural examination, the three-dimensional T1-weighted axial MRI sequence with the following parameters was used: 1.900 ms repetition time, 3.4 ms echo time, flip angle = 15, and a voxel size of 1.0 × 1.0 × 1.0 mm. GM, white matter, and the volume of the CSF were determined using SPM 8 with the VBM toolbox for spatially normalization by the means of high-dimensional DARTEL, bias correction, and segmentation into the different tissue classes of the T1 images. Intracranial volume (ICV) was calculated as the sum of GM, white matter, and CSF.

### MRI pattern classification

The SPARE-AD is an index previously developed using a support vector machine classifier that allows distinguishing between atrophy patterns in regions typically affected by clinical AD. It predicts the transformation from normal cognition to mild cognitive impairment and further on to clinical AD. Furthermore, we included in this study the SPARE-BA index to capture brain aging patterns of atrophy. The method of SPARE-BA has been described in more details earlier^9^.

### Statistical analysis

In 99 subjects, sNSE was not measurable, thus data about sNSE levels were available for *N* = 901 participants. To detect sex-dependent differences on a descriptive level, we divided the sample in females and males. To detect BMI-dependent differences on a descriptive level, we divided the sample in two groups. The overweight group contained participants with a BMI ≥25 kg/m², the normal-weighted group subjects with a BMI <25 kg/m². The *χ* ^2^ test was used to evaluate differences between groups for categorical variables (e.g., sex or medication) and the independent samples *t* test to compare means of continuous data (e.g., age or sNSE concentrations). Associations between sNSE levels and age, sex, BMI, vascular risk factors, or GMV were performed using linear regression analyses with robust estimates with STATA/MP version 13.1 (StataCorp, TX 77845, USA). Bootstrap analyses with 1,000 replicates were used to evaluate the robustness of the models. This did not change the results of calculations of the association between sNSE and age, specifically the sex-separated analyses (*p* = 0.008), nor the results of calculations of a non-linear association between sNSE, BMI, and WC (BMI *p* = 0.0056, WC *p* = 0.0049). In calculations concerning an association between sNSE and BMI, the non-linear gave a better model fit (*R* ^2^ increase from 2.2 to 2.9%). Thus, we included cubic splines for BMI. Statistical significance was defined as *p* < 0.05. Hypertension was defined by a systolic blood pressure (BP) ≥140 Hg and/or a diastolic BP ≥90 and/or antihypertensive intake. We further tested for associations between sNSE levels and SPARE-BA or SPARE-AD. Linear regression analyses with robust estimates on the dependent variable sNSE or GMV were performed. The analyses were adjusted for age and sex as basic confounders. As we observed an interaction term between age and sex, we included this interaction term in all further regression models. Analyses for GM were additionally adjusted for ICV and analyses for blood lipids and blood pressure were additionally adjusted for medication. Tests for non-linearity were performed graphically using lowess-smoothing plots for the full sample as well as sex-separated to assess possible interaction effects with sex. Analyses for blood pressure, hypertension, and blood lipids were controlled for medication. Specifically, all calculations were adjusted for age, sex, and age × sex interaction. Calculations on triglycerides, LDL-C, HDL-C, total cholesterol were additionally adjusted for lipid-lowering drugs. Calculations on systolic and diastolic blood pressure were additionally adjusted for antihypertensives.

## Results

### Sample characteristics

In total, we included 901 subjects (499 (55.4%) female) for the main analysis (Supplementary Table 1).

**Fig. 1:**
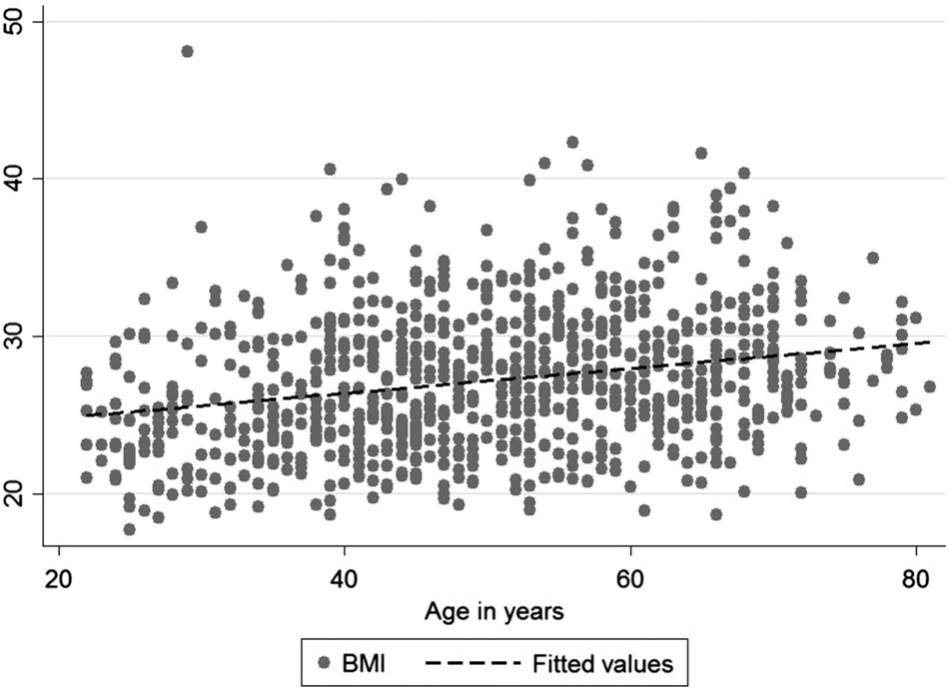
Increasing patterns of BMI during aging Subjects show an increasing BMI with advancing age. Linear trend line, no adjustment. BMI in kg/m²

Table 1 shows the descriptive statistics of sex-dependent differences in the sample. Compared with males, females had lower GMV, BMI, WC, systolic and diastolic BP, hypertension, and triglycerides, but higher HDL-C and total cholesterol on a descriptive level. The difference between males and females regarding smoking nicotine was also significant: Females were more often current smokers, whereas males were more often former smokers. No differences were seen for age, NSE, LDL-C, antihypertensive and lipid-lowering drugs in the group comparisons. Table 2 shows the descriptive analysis of BMI-dependent differences in the sample. Men were more often overweight than women were, and overweight subjects were older compared to individuals with normal weight (*p* < 0.001). Figure 1 shows a scatter plot with the linear increase of BMI during aging. GMV was lower in the overweight group (*p* = 0.004) in a subcohort of 832 subjects with MRI assessment. Obesity-associated comorbidities as hypertension and increased blood lipids were more often in the overweight group (*p* < 0.001), as well as antihypertensive intake (*p* < 0.001). Apart from HDL-C, all blood lipids (triglycerides, LDL-C, and total cholesterol) and lipid-lowering drug intake were higher in the overweight group (*p* < 0.001). Former smokers were more often in the obese group. Overweight showed somewhat lower sNSE levels than normal weighted, but the difference was not significant.

**Table 1:** Descriptive sample characteristics: group comparison of females and males

	Females *N*/mean	±SD	Males *N*/mean	±SD	*p* value
Participants	499		402		
Age (y)	50.45	±13.09	50.16	±14.16	0.7449
NSE (µg/L)	8.73	±3.81	8.95	±3.57	0.3739
GMV (cm^3^)*	557.77	±55.66	606.1	±65.68	**<0.001**
BMI (kg/m²)	26.83	±4.86	27.7	±3.68	**0.0029**
WC (cm)	82.85	±11.71	94.09	±10.95	**<0.001**
WC ≥88 (cm), women/≥102 (cm) men	379		315		**0.027**
Systolic BP in mmHg	118.83	±15.59	131.2	±15.25	**<0.001**
Diastolic BP in mmHg	74.35	±15.59	79.21	±9.89	**<0.001**
Hypertension	178		179		**<0.001**
*Smoking nicotine*					**<0.001**
Current	254		130		
Former	141		184		
Never					
Triglycerides in mmol/L	1.28	±0.67	1.58	±1.03	**<0.001**
LDL-C in mmol/L	3.4	±0.9	3.44	±0.88	0.5386
HDL-C in mmol/L	1.61	±0.36	1.32	±0.3	**<0.001**
Total cholesterol in mmol/L	5.59	±1.04	5.37	±1.03	**0.0022**
Antihypertensives	153		109		0.137
Lipid-lowering drugs	34		36		0.143

*BMI* body mass index, *BP* blood pressure, *GMV* gray matter volume, *HDL-C* high-density lipoprotein cholesterol, *LDL-C* low-density lipoprotein cholesterol, *NSE* neuron-specific enolase, *WC* waist circumference, *y* years

*Data available for 832 subjects; bold values represent statistical significant *p-*values defined as *p* < 0.05

**Table 2:** Descriptive sample characteristics: group comparisons in terms of the BMI

	BMI <25 *N*/mean	±SD or (%)	BMI ≥25 *N*/mean	±SD or (%)	*p* value
Participants	302	(33.5)	599	(66.5)	
*Sex*					**<0.001**
Women	203	(40.7)	296	(59.3)	
Men	99	(24.6)	303	(75.4)	
Age (y)	44.95	±13.4	53.0	±12.8	**<0.001**
NSE (µg/L)	9.0	±3.6	8.8	±3.7	0.407
GMV (cm^3^)*	590.4	±66.5	576.8	±63.2	**0.004**
WC (cm)	76.2	±7.2	93.7	±10.6	**<0.001**
WC ≥88 (cm), women	1	(0.7)	147	(99.3)	**<0.001**
WC ≥102 (cm), men	0	(0.0)	104	(100.0)	**<0.001**
Systolic BP in mmHg	116.4	±13.9	128.4	±16.4	**<0.001**
Diastolic BP in mmHg	72.4	±8.2	78.6	±9.6	**<0.001**
Hypertension	45	(12.6)	312	(87.4)	**<0.001**
*Smoking nicotine*					**0.013**
Current	72	(23.8)	118	(19.7)	
Former	89	(29.5)	236	(39.4)	
Never	140	(46.4)	244	(40.7)	
Triglycerides in mmol/L	1.0	±0.4	1.6	±1.0	**<0.001**
LDL-C in mmol/L	3.1	±0.9	3.5	±0.9	**<0.001**
HDL-C in mmol/L	1.6	±0.4	1.4	±0.3	**<0.001**
Total cholesterol in mmol/L	5.3	±1.0	5.6	±1.0	**<0.001**
Antihypertensives	33	(12.6)	229	(87.4)	**<0.001**
Lipid-lowering drugs	6	(8.6)	64	(91.4)	**<0.001**

*BMI* body mass index, *BP* blood pressure, *GMV* gray matter volume, *HDL-C* high-density lipoprotein cholesterol, *LDL-C* low-density lipoprotein cholesterol, *NSE* neuron-specific enolase, *WC* waist circumference, *y* years

*Data available for 832 subjects; bold values represent statistical significant *p*-values defined as *p* < 0.05

### Association between sNSE with age

**Fig. 2:**
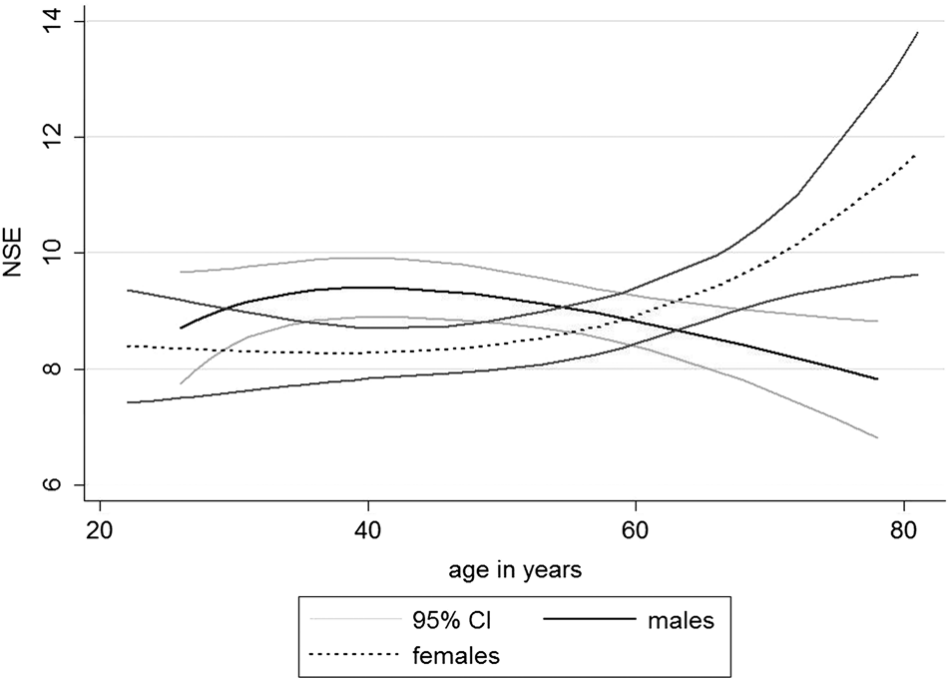
Non-linear age-dependent sNSE levels in men and women Women show constant low sNSE levels in younger years and increasing levels in higher ages, whereas men show increasing sNSE levels in young adulthood and a decrease in older ages. About an age of 60 years, sNSE values are similar in men and women. Sex-specific differences in the hormonal balance may serve as explanatory approach. After menopause, women have both lower levels of estradiol and testosterone. The missing estradiol-mediated neuroprotective effects may result in increased neural death, showing in increased sNSE levels. sNSE in µg/L

We did not find linear association between age and sNSE levels (*p* = 0.205, beta = 0.0122) (Table 3). As we observed different age effects in males and females, we included sex-stratified cubic splines for age (four knots equally placed; males: 26, 44, 57, 72 and females: 28, 45, 56, 71) and included an age × sex interaction term. We observed a significant age–sex interaction (*p* = 0.009). The levels of sNSE in aging are depicted as scatter plots separately for males and females in Supplementary Fig. 1. In sex-specific analyses, the association between age and sNSE was not significant (*p* = 0.1052) for males, whereas females showed a significant association between age and sNSE levels (*p* = 0.0363). For males, a U-shaped and for females a J-shaped relation between age and sNSE was observed (Fig. 2).

**Table 3:** Association of sNSE levels with age (non-linear), sex, and sex × age interaction

	Beta	SE	*T*	*p* value
Age (y)*^#^			*F* = 1.05	0.37
Sex*	−0.221	0.2467	−0.9	0.371
Age × sex interaction	0.05	0.019	2.64	0.009

*sNSE* serum neuron-specific enolase

*Unadjusted

^#^Variables were treated non-linear as splines; no betas, SE possible

### Association between sNSE and BMI

**Fig. 3:**
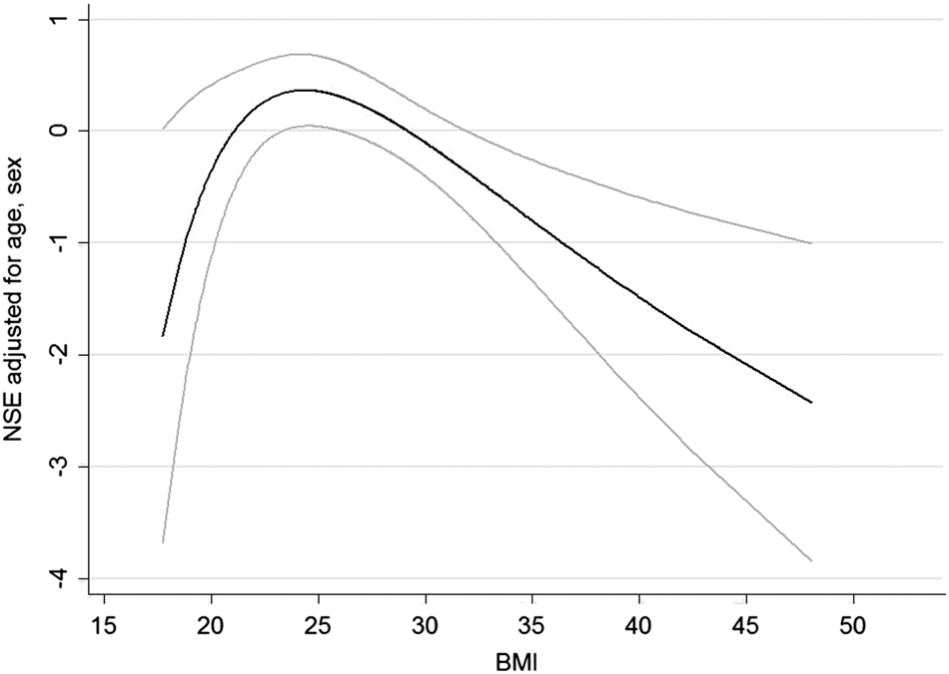
Non-linear association between sNSE levels and BMI. sNSE levels increase up to a BMI of >25, and decline with higher BMI values Obesity-associated neuronal damage may reflect the increasing sNSE levels up to a BMI <25. In subjects with a very high BMI (>25), the excess body fat affected cerebral structures over a long time, what could result in decreased GMV. This reduced GMV could show in a drop of sNSE levels, as there would be less and less GMV containing NSE. Other explanatory approaches for decreasing sNSE levels at BMI levels >25 are impairments in glucose metabolism and neuronal differentiation. NSE in µg/L

sNSE levels decreased with increasing BMI in the linear model (Supplementary Fig. 2). In the non-linear model, sNSE values and BMI showed a U-shaped negative association (Supplementary Fig. 3). These effects were significant for both, BMI (*p* = 0.0056) and WC (*p* = 0.0042), apart from an influence of age and sex. Specifically, sNSE levels increased with higher BMI values up to a BMI of 25 kg/m² and decreased thereafter (Fig. 3). *p* values for the association between sNSE levels and BMI or WC were similar; therefore, we performed the following analyses with BMI only. To investigate presumed age-dependent differences in overweight-associated cerebral impairment, we stratified the sample for age. Regarding a possible interaction between BMI and age, the interaction term was not significant (*p* = 0.244), but when stratifying the sample into different age groups (age <40, age 40–60, age >60), only the oldest sample revealed a significant association between sNSE and BMI (*p* = 0.02). When regarding the overweight and normal-weighted separately, a significant association between sNSE levels and BMI was specific for overweight elderly (*p* = 0.0365). Concerning hypertension, blood pressure, blood lipids and smoking status, only HDL (*p* = 0.016), and current smoking (*p* = 0.019) exhibited a significant inverse association with sNSE in the full sample (Table 4).

**Table 4:** Associations of sNSE levels with BMI, WC, and lipids

	Beta	SE	*T*	*p* value
BMI (kg/m^2^)^#^			*F* = 4.22	**0.0056**
WC (cm)^#^			*F* = 4.44	**0.0042**
Triglycerides in mmol/L*	−0.19	0.1379	−1.38	0.168
LDL-C in mmol/L*	0.1076	0.152	0.71	0.479
HDL-C in mmol/L*	0.8991	0.3734	2.41	**0.016**
Total cholesterol in mmol/L*	0.1611	0.1369	1.18	0.24
Lipid-lowering drugs^1^	−0.0684	0.4902	−0.14	0.889
*Smoking nicotine*				
Current (reference)				
Former	−0.2799	0.2903	−0.96	0.335
Never	−0.7749	0.3309	−2.34	**0.019**

*BMI* body mass index, *HDL-C* high-density lipoprotein cholesterol, *LDL-C* low-density lipoprotein cholesterol, *sNSE* serum neuron-specific enolase, *WC* waist circumference

Associations adjusted for age (non-linear), sex, and age × sex interaction

^#^Variables were treated non-linear as splines; no betas, SE possible

*Additionally adjusted for lipid-lowering drugs; bold values represent statistical significant p-values defined as *p* < 0.05

### Association between sNSE and GMV, SPARE-BA and SPARE-AD

sNSE levels were not associated with GMV (*p* = 0.963) after adjustment for ICV (Table 5). Stratifying the sample into subjects with and without overweight parallel to Mueller et al., results were also not significant (BMI <25: *p* = 0.854; BMI ≥25: *p* = 0.416). Overweight elderly aged >60 years, showed a negative association with GMV, yet the association was not significant. In normal-weighted elderly, this tendency was not seen (BMI <25: *p* = 0.65; BMI ≥25: *p* = 0.068). Also no significant associations with sNSE and SPARE-BA (*p* = 0.928) or SPARE-AD (*p* = 0.643) were observed in the total sample or in elderly aged >65 years.

**Table 5:** Association of sNSE levels with GMV and SPARE-BA and SPARE-AD

	Beta	SE	*T*	*p* value
GMV*	−0.0116	0.2462	−0.05	0.963
SPARE-BA	−0.0001	0.0106	−0.09	0.928
SPARE-AD	0.0045	0.0097	0.46	0.643

*GMV* gray matter volume, *sNSE* serum neuron-specific enolase, *SPARE-AD* spatial patterns of abnormality for recognition of early Alzheimer’s disease, *SPARE-BA* spatial pattern of atrophy for recognition of brain aging, *WC* waist circumference

Associations adjusted for age (non-linear), sex, and age × sex interaction

GMV, SPARE-AD/BA treated as outcome

*Additionally adjusted for ICV, data available for 832 subjects

However, SPARE-BA and SPARE-AD were associated with GMV (*p* < 0.001).

## Discussion

We showed sex-dependent differences in the patterns of sNSE levels with increasing age, with an increase of sNSE levels in elderly women.

sNSE levels and BMI were non-linearly associated, showing a parabolic association with decreasing sNSE levels at BMI values >25 kg/m².

We could not substantiate the hypothesis of an inverse association between sNSE levels and GMV, yet we saw the tendency in elderly subjects with overweight. Atrophy patterns in brain aging (SPARE-BA) or for AD-like patterns of atrophy (SPARE-AD) were not associated with sNSE levels.

### Association between sNSE and age

The effect of age on NSE concentrations is differently discussed in the literature, partly because NSE levels may be assessed in CFS or in serum^13, 21, 22^. Until now, only NSE levels in CSF and age were positively associated^22^. In contrast, serum NSE levels showed no age-dependent alterations in two studies with *N* = 108 and *N* = 41 probands^13, 21^. Corresponding to previous studies based on sNSE levels^13, 21^, we report no significant linear association between NSE and age. Considering the extensive neural loss in the course of AD, studies have aimed to show disease-associated alterations in sNSE levels. However, studies produced inconsistent results concerning sNSE levels in AD^23, 24^. Chaves et al.^23^ found no differences in sNSE levels between AD patients and healthy elderly controls.

However, we did find significant non-linear sex-specific associations with aging. Specifically, females showing augmented, males decreasing sNSE levels associated with aging. We saw no association between sex and sNSE levels, which is in line with previous studies^13^, but an interaction between sex and age on sNSE levels. It seems contradictive that there was no association between sex and sNSE levels, but an association between sNSE levels and sex depending on age. Specific differences between the sNSE levels of men and women may only show with advancing age. Therefore, no association would be found when regarding participants of all ages, for the differences between males and females would be leveled.

To our knowledge, our study is the first to describe a divergence of age-dependent sNSE levels in women and men in a large sample from the general population (*N* = 901). One may speculate sex-specific differences in the hormonal balance may serve as an explanatory approach. However, currently no research has further explored this association. Levels of sex steroids as estradiol and testosterone decline with age in both sexes. After menopause, estradiol levels show sharp descends in women, whereas testosterone levels decline more gradually in men. Elderly women have both lower levels of estradiol and testosterone^25^, accompanied by higher levels of cholesterol. This may be explained by increasing NSE levels, as enolases mobilize cholesterol^26^.

There is growing evidence for estradiol mediating neuroprotective effects in the hippocampus^27^. Since NSE is related to hippocampal structures, a possible explanation for our results is that the decline of estradiol-mediated neuroprotective effects is accompanied by accelerated neuronal death eventually resulting in increased sNSE levels.

### Association between sNSE and BMI

Our hypotheses of a positive association between sNSE levels and BMI was based on the study of Mueller et al^14^. They described an inverse correlation between GM density in cerebellum and hippocampus and sNSE levels in *N* = 27 overweight subjects. They hypothesized that increased sNSE levels are a result of obesity-associated structural damage of GM. Accordingly, obesity-associated neuronal damage could reflect our findings of increasing sNSE levels up to a BMI <25. Moreover, the trend towards higher sNSE levels in overweight subjects in our study confirms the findings of Mueller et al. They described sNSE levels to be in the reference range in all subjects, but the overweight showed levels near the upper limit (18.3 µg/L)^14^.

We report a parabolic association between sNSE levels and BMI, with decreasing sNSE levels at BMI values >25. Moreover, in higher ages only overweight subjects showed a negative association with sNSE levels. Yet, we could not see the tendency in young adults, as depicted in the sample of Mueller et al. This leads to the assumption, that there may be age-dependent differences concerning an association between sNSE levels in overweight subjects.

### Association between sNSE and GMV, SPARE-BA and SPARE-AD

We did not see a linear association between GMV or SPARE-BA or SPARE-AD sNSE levels in our sample. As a marker for neuronal injury, elevated sNSE levels result from an actual cerebral destruction, especially from GM damage. sNSE levels have shown to correlate positively with the severity level of the head injury, and therefore with the extent of brain cell damage^28^. It could be assumed, in subjects with a very high BMI, the excess body fat affected cerebral structures over a long time, resulting in decreased GMV. This reduced GMV could show in a drop of sNSE levels, as there would be less and less GMV containing NSE. Decreased sNSE levels have been reported in higher extent of brain atrophy in AD^23^, accordingly similar dynamics of sNSE levels can be expected in an obesity-related negative association with GMV.

The sample of Mueller et al. contained young adults (average age in the overweight group 26.4 ± 5.4 years), whereas in our sample the average age was in middle adulthood (average age in the overweight group 53.0 ± 12.8). Thus, the detrimental effects of body fat could not have affected the brain for a long time. Further, we have seen a non-significant association between sNSE levels and GMV in obese elderly, but not in non-obese subjects, which is also in agreement with the findings of Mueller et al^14^. The study of Mueller et al.^14^ stated, that the higher the BMI, the greater the loss of GM density, comparable with mild cognitive impairment in the elderly. An induction of progressive brain alterations in obese subjects might be discussed, as studies revealed obesity-associated increased oxidative stress^29^, and chronic inflammation^30^.

Furthermore, decreased sNSE levels could result from alterations in the glucose metabolism: As glycolytic enzyme, NSE is directly involved in the metabolism of glucose and obesity-related impairment in glucose metabolism in the frontal cortex have been reported^31^.

Another explanatory approach is neuronal differentiation. NSE is a specific marker for mature nerve cells, and is closely correlated to the differentiated state^12^. Depending on tissue and development state, different combinations of the subunits are expressed. γγ is specific for mature neurons with full synaptic connections, whereas during neuronal migration, hybrid enolases (αγ) are found (non-neuronal enolase)^32^. Obesity-related impairments on hippocampal neurogenesis have been demonstrated in animal studies with mice receiving a high-fat diet^33, 34^. As NSE is associated with hippocampal structures, it can be speculated that obesity-related alterations of neuronal maturation and neurogenesis extend to the development of NSE.

In conclusion, we propose in accordance to previous studies an actual neuronal damage in the early stages of obesity. However, with progression of overweight, we assume more profound effects of excess body fat to the brain.

Due to the cross-sectional design, we cannot investigate if the alterations in sNSE levels precede the onset of obesity or are resulting from the adverse effects of an enhanced bodyweight. As one study showed that a high BMI in mid-life was associated with temporal atrophy 24 years later^35^ and NSE is an indicator for neuronal injury, the current data support the second hypothesis.

### Strength and limitations

Strength of this study is the cross-sectional population-based sample. Thus, we can assume a sample, which is representative for the population. Stratification by age adds valuable information specific for particular age groups. With consideration of pattern classifiers in brain atrophy, we are able to make statements concerning the impact of average or increased aging processes on sNSE levels.

We measured height and weight by research assistants, therefore, we can rule out false statements concerning the BMI. As we performed analyses with BMI and WC, we can preclude effects that are unique for one measurement of obesity. We did not include data for deficits in glucose metabolism, thus this consideration should be examined in further research. To prevent confounding, we controlled for medication. However, we cannot rule out other confounding factors that may have affected the calculations.

## Conclusions

Our study presents an analysis of a well-described cohort with a large number of participants, providing valuable and novel insights into alterations of sNSE levels in aging and obesity. We were able to show that sNSE levels and obesity are nonlinearly associated, even after adjusting for important confounders. Moreover, only in women, age was associated with increasing sNSE levels, thus we assume sex-specific pathomechanisms. With the determination of NSE levels in serum, we provide a valuable, easy determinable marker to get insights into brain-specific cellular alterations. Future studies, focusing on cerebral manifestations of obesity and the effect of their therapy would help to get to better and more individualized therapy strategies and to prevent or even undo cognitive impairment.

## Electronic supplementary material

Supp_Table.1

Supp_Fig.1

Supp_Fig.2

Supp_Fig.3

Supp_Fig.4

Supp_Fig.5
