# Supplementary material for: Association between serum neuron-specific enolase, age, overweight, and structural MRI patterns in 901 subjects
Source: Transl Psychiatry. 2017 Dec 8;7:1272. doi: 10.1038/s41398-017-0035-0 (PMC5802579; doi:10.1038/s41398-017-0035-0)
Supplement: Supplementary file 1 — Supp_Table.1 [file 41398_2017_35_MOESM1_ESM.docx]

Supplement Table 1 Descriptive sample characteristics

|  | N = 901  Mean/N | ± SD or (%) |
| --- | --- | --- |
| Age (y) | 50.3 | ± 13.5 |
| Men/women | 402/499 |  |
| BMI (kg/m²) | 27.2 | ± 4.3 |
| BMI >25 (kg/m²) | 598 | (66.4) |
| BMI ≥30 (kg/m²) | 222 | (24.6) |
| BMI >35 (kg/m²) | 42 | (4.7) |
| WC ≥88 cm (women) | 148 | (29.7) |
| WC ≥102 cm (men) | 104 | (25.9) |
| NSE (µg/L) | 8.8 | ± 3.7 |
| GMV (cm^3^) * | 579.3 | ± 64.9 |
| Systolic BP in mm Hg | 124.4 | ± 16.6 |
| Diastolic BP in mm Hg | 76.5 | ± 9.6 |
| Hypertension | 357 | (39.6) |
| Smoking nicotine |  |  |
| Current | 190 | (21.1) |
| Former | 325 | (36.1) |
| Never | 384 | (42.6) |
| Triglycerides in mmol/L | 1.4 | ± 0.9 |
| LDL-C in mmol/L | 3.4 | ± 0.9 |
| HDL-C in mmol/L | 1.5 | ± 0.4 |
| Total cholesterol in mmol/L | 5.5 | ± 1.0 |

* Data available for 832 subjects

Abbreviations: BMI, body mass index; BP, blood pressure; cm, centimeter; GMV, gray matter volume; HDL-C, high density lipoprotein cholesterol; kg/m², kilogram per square meter; LDL-C, low density lipoprotein cholesterol; mm Hg, millimeter of mercury; mmol/L, millimol per liter; µg/L, microgram per liter; N, number; NSE, neuron specific enolase; SD, standard deviation; WC, waist circumference; y, years
