# Supplementary figures and images for: Association between serum neuron-specific enolase, age, overweight, and structural MRI patterns in 901 subjects
Source: Transl Psychiatry. 2017 Dec 8;7:1272. doi: 10.1038/s41398-017-0035-0 (PMC5802579; doi:10.1038/s41398-017-0035-0)

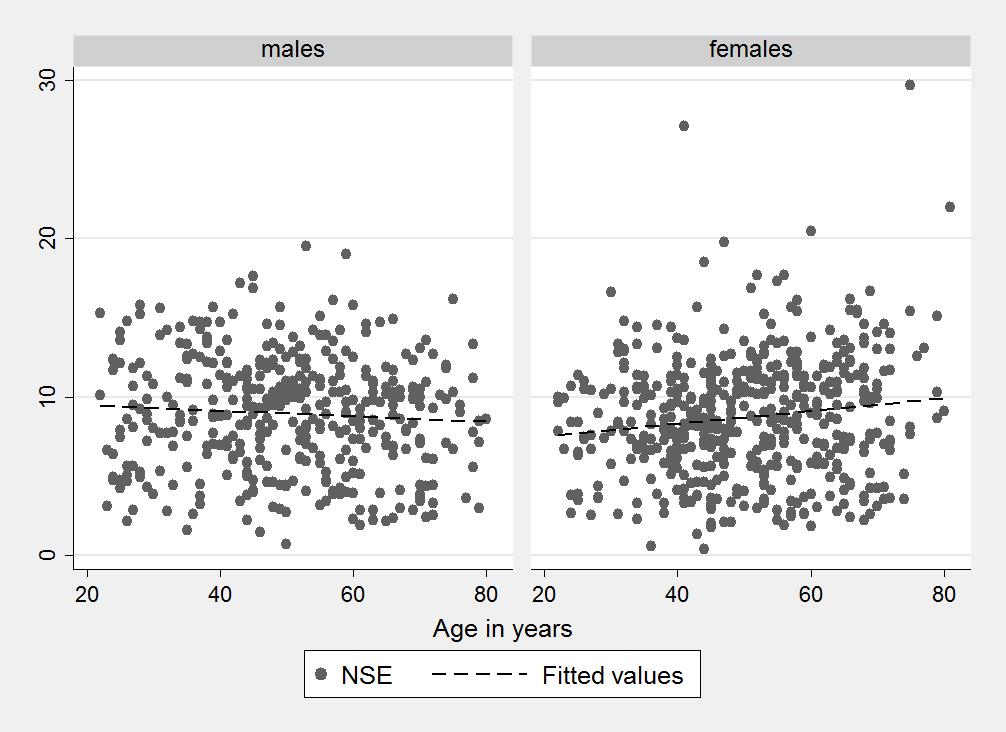

Supplement: Supplementary file 2 — Supp_Fig.1 [file 41398_2017_35_MOESM2_ESM.jpg]

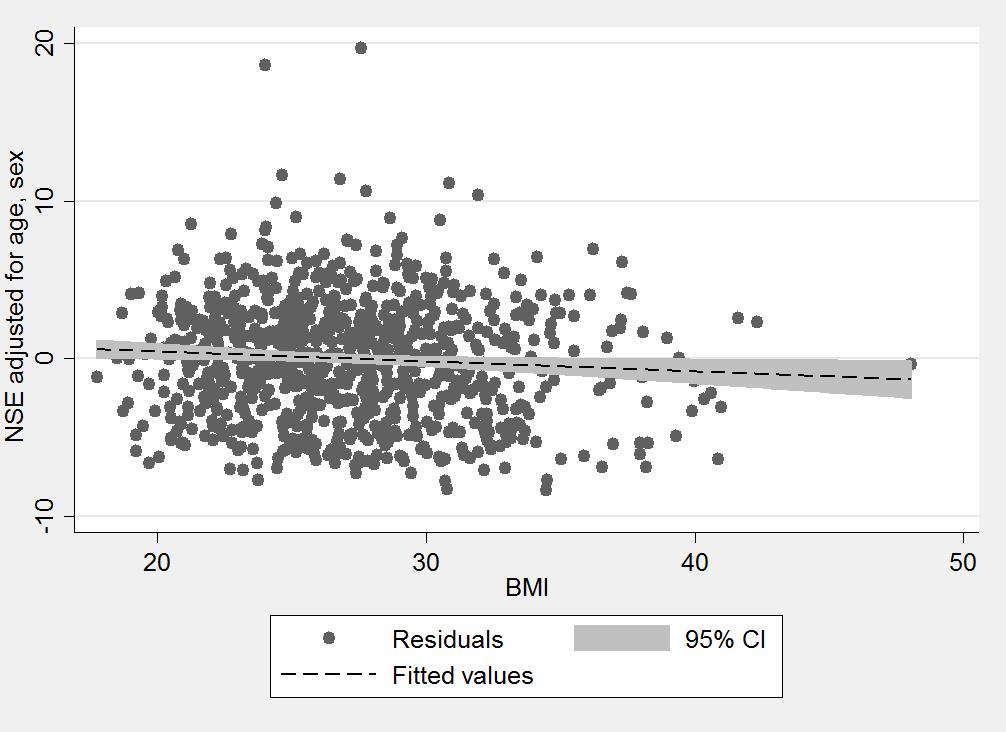

Supplement: Supplementary file 3 — Supp_Fig.2 [file 41398_2017_35_MOESM3_ESM.jpg]

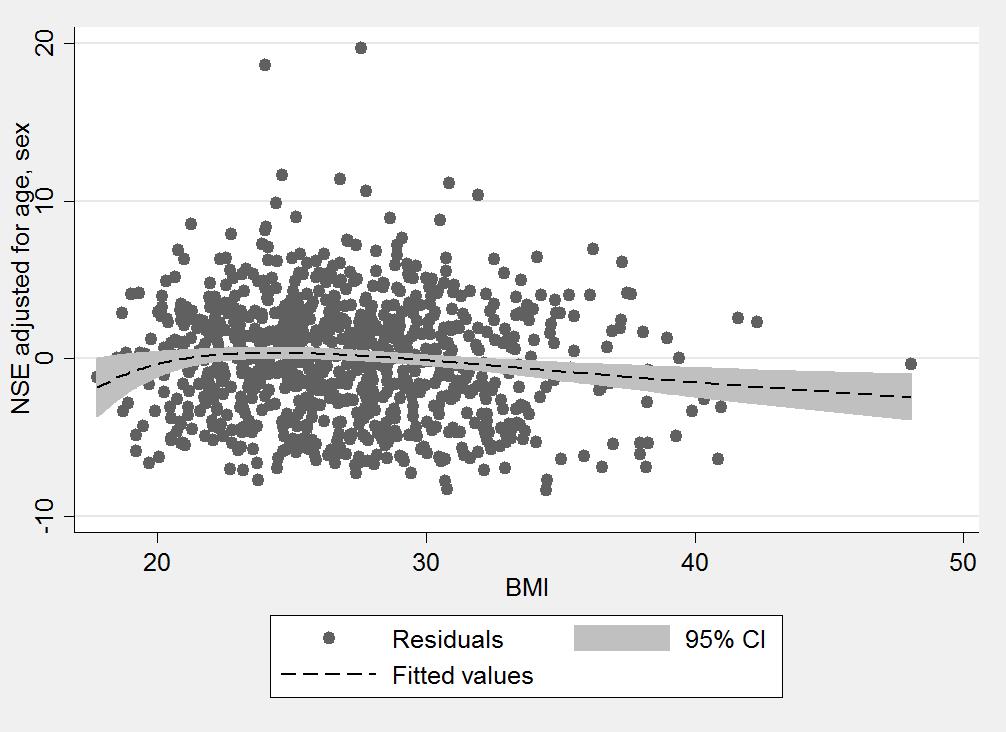

Supplement: Supplementary file 4 — Supp_Fig.3 [file 41398_2017_35_MOESM4_ESM.jpg]
