# Supplementary material for: Association between serum neuron-specific enolase, age, overweight, and structural MRI patterns in 901 subjects
Source: Transl Psychiatry. 2017 Dec 8;7:1272. doi: 10.1038/s41398-017-0035-0 (PMC5802579; doi:10.1038/s41398-017-0035-0)
Supplement: Supplementary file 5 — Supp_Fig.4 [file 41398_2017_35_MOESM5_ESM.docx]

Supplement Fig. 4 Flowchart for exclusion criteria of sNSE measurement and MRI of the brain

1,000 subjects, aged 20-79 years at the time of baseline examination

sNSE not measureable (99 subjects excluded)

901 subjects

MRI quality control (69 images excluded)

832 subjects

N = 832 subjects with MRI of the brain and measurement of sNSE levels

In N=1000 subjects, aged 20-79 years at the time of baseline examination, MRI of the brain was performed. In N=99 subjects sNSE was not measureable, leaving N=901 subjects with information about sNSE values and MRI of the brain. 69 MRI images failed quality control and were excluded. Finally, in N=832 subjects information about sNSE levels and MRI of the brain were available. sNSE, serum neuron specific enolase
