# Supplementary material for: Association between serum neuron-specific enolase, age, overweight, and structural MRI patterns in 901 subjects
Source: Transl Psychiatry. 2017 Dec 8;7:1272. doi: 10.1038/s41398-017-0035-0 (PMC5802579; doi:10.1038/s41398-017-0035-0)
Supplement: Supplementary file 6 — Supp_Fig.5 [file 41398_2017_35_MOESM6_ESM.doc]

Supplement Fig. 5. Flowchart for exclusion criteria of MRI of the brain in SHIP-TREND

4420 subjects, aged 20-83 years at the time of baseline examination in SHIP-TREND

without MRI scanning

2186 subjects

without MRI of the brain

2154 subjects

exclusion of subjects with neurological disorders

2010 subjects

MRI quality control

1934 subjects

VBM 8 toolbox homogeneity check

1586 subjects

N = 1586 subjects with MRI of the brain in SHIP-TREND

Subjects who fulfilled exclusion criteria against MRI (e.g. pregnancy, cardiac pacemaker) or who refused participation were excluded (remaining N = 2186 subjects). T1-weighted MRI images of the brain were available for 2154 subjects of SHIP-TREND. Subjects with a stroke, Parkinson’s disease, epilepsy, hydrocephalus, enlarged ventricles, pathological lesions, history of cerebral tumour and multiple sclerosis were excluded from the present analyses (remaining N = 2010 subjects). Moreover, we excluded images with severe inhomogeneities of the magnetic field or strong movement artefacts (remaining subjects N = 1934).Within the VBM 8 toolbox homogeneity check was conducted (remaining subjects N = 1586).
